# Prevalence of Intestinal Parasitic Infections among Mentally Disabled Children and Adults of Urmia, Iran

**Published:** 2010-06

**Authors:** Kh Hazrati Tappeh, H Mohammadzadeh, R Nejad Rahim, A Barazesh, Sh Khashaveh, H Taherkhani

**Affiliations:** 1 Dept. Of Parasitology & Mycology, Faculty of Medicine, Urmia University of Medical Sciences, Urmia, Iran; 2 Infectious disease specialist, Dept. Of Infectious Disease, Taleghani Hospital, Urmia Medical University, Urmia, Iran; 3 Dept. Of Parasitology, Faculty of Medicine, Golestan Medical University, Gorgan

**Keywords:** Intestinal parasites, Mental Retardation, Prevalence, Iran

## Abstract

**Background:**

The prevalence of intestinal parasites infection in institutions for mental retardation of Urmia City, West Azerbaijan Province, Iran was investigated.

**Methods:**

This descriptive - cross sectional study was carried out in institutions of mentally retarded patients of Urmia city in 2007–2008. Fecal samples of 225 less than 29 year old mentally disabled individuals were examined using direct smear, formalin - ether concentration. Beside their scotch tape samples were observed for *Enterobius* eggs. Statistical evaluation was performed by SPSS 10.

**Results:**

Of 225 mentally retarded persons, 118(52.4%) and 107(47.6%) were female and male. The overall prevalence of infection was 20.4% and that of male, and female were 20.5% and 20.3%, respectively. 17.3% of examined individuals had protozoa infection and 3.1% showed *Enterobius vermicularis* eggs. The infection rates of detected intestinal protozoa were *Entamoeba coli* 9.7%, *Giardia lamblia* 6.2%, *Iodoamoeba butschlii* 5.7%, *Blastocystis hominis* 4%, and *Entamoeba histolytica/dispar* 0.4%. Forty percent of 1–5 year, 22.8% of 6–14 year, 22.2% of 15–18 year, and 16.8% of more than 18-year age groups, had positive results in their tests. According to IQ test results, 23.8% of less than 25 score group, 19.6% of 25–50, 17.2% of 50–75, and 40% of 75–90 groups were infected.

**Conclusion:**

More efforts for increasing sanitation level and prompt diagnosis and treatment of infected persons in these institutions are necessary.

## Introduction

Parasitic diseases have great impact on life quality of people all over the world especially in developing countries. Five out of 6 seasonal bulletins of WHO are about parasitic infections. Actually, the prevalence of parasitic infections in a particular region depends not only on bioenvironmental situation, but also on social, economical, and cultural conditions. In developing countries that are mainly situated in tropical areas, lack of access to health services, malnutrition, and poor sanitation, increase vulnerability to infection (1).

Because of physiological and immunological reasons children are more susceptible to parasitic diseases that may have deteriorating effects of their physical and mental growth. In addition, age plays an important role in *Giardia lamblia*, *Enterobius vermicularis* and *Hymenolepis nana* infections (2).

Intestinal parasites can cause a variety of signs and symptoms in infected persons. Institutions, in which people live in crowd and for long time with each other, particularly when sanitary level is low, are suitable environments for occurrence and transmission of these parasites. Such conditions are more likely to be seen in rehabilitation centers, and higher prevalence rate of contagious parasitic infections in their habitants is predictable and so it is necessary to do investigations on these groups for intestinal parasitic infections (3). The aim of this study was to find the prevalence of intestinal parasites among mentally retarded patients in Urmia, Iran.

## Materials and Methods

This descriptive - cross sectional study with total population sampling was carried out on 225 mentally retarded children and adults who were living in private and governmental rehabilitation institutions of Urmia city, West Azerbaijan Province, Iran.

Descriptive meetings were arranged and necessary co-ordinations for collecting stool samples and scotch tapes were done. Individual questionnaires, containing personal information such as name, family name, age, sex and the IQ test score were filled. Two fecal and two scotch tape samples were collected from each patient in first and third days and transferred to the Parasitology Lab of Urmia Faculty of Medicine. Stool samples were examined using usual methods including direct wet smear with saline and lugol's iodine solution, and formalin-ether concentration, all based on the protocol of World Health Organization (4). The slides of scotch tapes were investigated under X10 and X40 lenses of light microscope.

The results were analyzed using SPSS software. Written permissions had been obtained from Office of Research Affairs in Urmia University of Medical Sciences and Welfare Organization of Urmia, Iran.

## Results

Of 225 mentally retarded persons, 2.2% aged 1–5, 40.8% aged 6–14, 12% aged 15–18, and 44.8% aged >18 years. 52.5% were female. IQ test scores were below 25 for 29.1%, 25–50 for 28.2%, 50–75 for 40.2% and 75–90 for 2.3% of patients.

20.5% of cases were infected with intestinal parasitic infection. Seven patients (3.1%) showed eggs of *E. vermicularis* and 39 (17.3%) had protozoan parasites. No helminth infection other than *Enterobius* was found.

**Fig. 1:**
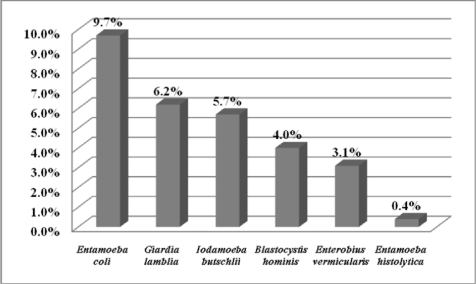
Prevalence of intestinal parasitic infections among 225 mentally retarded patients in rehabilitation centers of Urmia, Iran

Five intestinal protozoan species were detected in examined fecal specimens with prevalences shown in Fig. 1. Just one infected patient with *Entamoeba histolytica*/*dispar* was found.

**Fig. 2:**
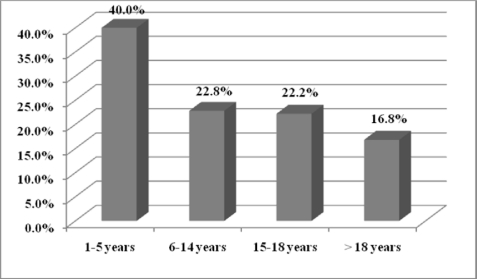
Prevalence of intestinal parasitic infection among age groups of 225 mentally retarded patients in rehabilitation centers of Urmia, Iran

Of 46 infected persons, 2 (4.3%), 21 (45.6%), 6 (13%), and 17 (36.9%) were in age groups of 1–5, 6–14, 15–18 and more than 18 years, respectively. Infection prevalence in every age group showed a decrease in infection rate on aging (Fig. 2).

Twenty four (20.3%) of females and twenty two (20.5%) of males were infected. IQ test scores for infected patients were below 25 for 34%, 25–50 for 27.2%, 50–75 for 34%, and 75–90 for 4.5%. Regardless of the last group (with just 5 members and 2 of them infected), infection rate showed a slight decrease with higher IQ scores (Table 1).

**Table 1:** Distribution of intestinal parasitic infections among IQ test scores of 225 mentally retarded patients in rehabilitation centers of Urmia, Iran

IQ test scores	Infected (Percent in IQ Group)	Non-infected (Percent in IQ Group)	Total
<25	15 (23.8)	48 (76.2)	63
25–50	12 (19.6)	49 (80.4)	61
51–75	15 (17.2)	72 (82.8)	87
76–90	2 (40)	3 (60)	5
Total	44	172	216

## Discussion

The prevalence of intestinal parasitic infection in 225 people of rehabilitation centers in Urmia was 20.5 percent. It is comparable with the results (23% infection in 550 mentally retarded people) obtained in Italy (5). Much lower infection rates in another study, in New York (7.3% infection) is reported (6). However, much higher results like studies in Korea (7) with 35.7% and Egypt (8) with 76.67% infection rates have also been reported.

Comparing with studies done in Philippine (9) and Hamadan city of Iran (10), the prevalence of *E. coli* is lower in Urmia institutions, but the infection rate of other intestinal protozoa including *G.lamblia*, *I.butschlii*, and *B.hominis* was higher.

Referring to Fig. 2, it can be seen (not proven statistically) that intestinal parasitic infections are most prevalent in lower ages, probably because of less immunity and educational levels.

A study on school attending children of Isfahan showed *G.lamblia* cysts in 19.3% of 228 students (11). In addition, a study in Thailand (12) on guardianless children revealed that 37.7% of them were infected with *Giardia*. In both studies, *Giardia* had the highest prevalence of intestinal protozoa whereas in our study it was the second one. As *G. lamblia* is mostly transmitted through water, it may be concluded that there may be no discrepancy between normal people and retarded people in *G.lamblia* infection. The prevalence of *B.hominis* in our study (4%) was lower than some studies carried out in other countries. In the previously mentioned study in Thailand, the prevalence of this organism was the highest in fecal sample of 106 guard less children and in another survey in Philippine (13), stools of 40.7% of 172 children had *Blastocystis*.

In this study, the prevalence of *E. vermicularis* was much less than the study done in Isfahan (20.7%), and a study in Izmir (14) in which 32.21% of 208 examined guardless children had enterobiasis and the survey in New York (6) on people living in retarded patients' institutions with the prevalence of 41.5% of *E.vermicularis*. It could be due to unsuitable sampling in Urmia.

Relatively low incidences of intestinal parasites especially helminthic infections achieved in this study is in accordance with the fact that in general the prevalence of intestinal worms appears to be becoming rarer in Iran (15). Nevertheless, parasites, more commonly the protozoa, yet are present, capable to produce mortality and morbidity and even to re-emergence if the public health system fails to continue its control and preventive measures. Thus, it is essential to maintain or intensify such measures in community and particularly for disabled people who are not able to protect themselves.
